# The Ecophysiological Response of Two Invasive Submerged Plants to Light and Nitrogen

**DOI:** 10.3389/fpls.2019.01747

**Published:** 2020-02-07

**Authors:** Sándor Szabó, Edwin T. H. M. Peeters, Gábor Borics, Szilvia Veres, Péter Tamás Nagy, Balázs András Lukács

**Affiliations:** ^1^Department of Biology, University of Nyíregyháza, Nyíregyháza, Hungary; ^2^Aquatic Ecology and Water Quality Group, Wageningen University, Wageningen, Netherlands; ^3^Department of Tisza River Research, Danube Research Institute, MTA Centre for Ecological Research, Debrecen, Hungary; ^4^Department of Agricultural Botany, Crop Physiology and Biotechnology, Institute of Crop Sciences, University of Debrecen, Debrecen, Hungary; ^5^Institute of Water and Environmental Management, University of Debrecen, Debrecen, Hungary; ^6^Wetland Ecology Research Group, Danube Research Institute, MTA Centre for Ecological Research, Debrecen, Hungary

**Keywords:** *Elodea*, growth rate, interaction, nutrient removal, photochemical efficiency

## Abstract

Two submerged *Elodea* species have small differences in their ecophysiological responses when exposed to individual environmental factors. However, field observations showed that under eutrophic conditions with low light availability, *Elodea canadensis* could be displaced by *Elodea nuttallii*. Here we investigated the combined effect of environmental factors on the ecophysiological response of the two species in order to explain the differences in their invasion successes. We cultivated the plants in aquaria containing five different nitrogen (N) concentrations and incubated at five different light intensities. For both species increasing nitrogen concentrations resulted in increased relative growth rate, chlorophyll concentration, and actual photochemical efficiency of photosystem II (Φ_PSII_), however, they produced less roots. Lowering light intensity resulted in a lower relative growth rate, root production, and nutrient removal. In contrast, chlorophyll concentration in the leaves, and Φ_PSII_ increased. The main difference between the two *Elodea* species was that the light compensation point (*I*_c_) and weight loss in the dark were significantly higher and photochemical efficiency and chlorophyll concentration were significantly lower for *E. canadensis* than for *E. nuttallii*, indicating that the latter can survive under much more shady and hypertrophic conditions. The change in nitrogen concentration of the media and in tissue concentration of the plants indicated that *E. nuttallii* has a higher nitrogen removal capacity. The ecophysiological differences between the two species can be an explanation for invasion success of *E. nuttallii* over *E. canadensis* and thus may explain why the latter is replaced by the first.

## Introduction

Light and nutrient availability are key factors governing the distribution and growth of submerged macrophytes in aquatic ecosystems (Chambers, 1987; Chambers and Kalff, 1987; Best et al., 2001). Eutrophication changes the availability of nutrients and light for submerged macrophytes and this might be beneficial for some macrophytes but harmful to others (Cao et al., 2011). Rooted submerged plants grow under relatively low light conditions at the start of the growing season, and their growth is accelerated when temperature rises. Due to their apical elongation, they grow towards the water surface and become exposed to higher light levels which is favorable for photon capture (Kuni, 1982). The relative competitive advantage of submerged macrophytes depends on their capacity to take up nutrients and to capture light (Szabó et al., 2010). The sooner a species reaches the higher light level, the better this species can shade out and finally displace others (Barrat-Segretain, 2005), and this is especially important in eutrophic and turbid waters. Light availability for submerged plants is not only influenced by shading of neighboring plants but also by the presence of free-floating vegetation (Scheffer et al., 2003; Morris et al., 2004; Lu et al., 2013; van Gerven et al., 2015). Floating algal bed, planktonic and periphytic algae can additionally reduce inherently poor light conditions under the water and lead to the decline of aquatic vegetation (Phillips et al., 1978; Hillebrand, 1983). Ultimately, plants that have a lower light compensation point or have a higher shade tolerance are expected to be better survivors in turbid water or under the shade of floating macrophytes. Consequently, eutrophication opens the window of opportunity for submerged macrophytes that have those traits (James et al., 1999). Frequently, species with those traits invade new regions where they are more competitive than native species (Vilà and Weiner, 2004; Espinar et al., 2015).

An interesting case is the introduction of congeneric exotic water plant species like *Elodea canadensis* and *Elodea nuttallii*. Both species are native to North America and were introduced to Europe. *E. canadensis* arrived to the British Isles in the middle of the 19th century. After the first records of *E. nuttallii* in 1966, this new invader spread rapidly all over England and displaced *E. canadensis* even at sites where the latter was well-established (Lund, 1979; Simpson, 1984; Simpson, 1990). This displacement was relatively rapid, taking place over a period of years, and is consistent with displacements elsewhere in Western Europe (Barrat-Segretain, 2001). From 1870 onwards *E. canadensis* was also present in Central European waters while *E. nuttallii* arrived in the beginning of the 21th century in Central European waters i.e. Slovakia (Ot'ahel'ová and Valachovič, 2002), Hungary (Sipos et al., 2003; Király et al., 2007), Croatia, (Grudnik and Germ, 2013; Grudnik et al., 2014; Kočic et al., 2014) and it was questionable whether *E. canadensis* would be displaced.

Several authors suggested that the displacement of *E. canadensis* by *E. nuttallii* was due to differences in their ecophysiological responses to environmental variables. However, observed differences in their ecophysiological responses like growth rate along a single gradient of temperature (Kuni, 1982; Kuni, 1984), nitrogen and phosphorus accumulation (Robach et al., 1995; James et al., 2006), photosynthesis and respiration (James et al., 1999), allelopathic activity against algae (Erhard and Gross, 2006; Lürling et al., 2006) all seemed too slight to induce such a displacement. Also, the tiny differences in life-history traits like fragment regeneration, colonization, and palatability seem also not plausible to explain the observed displacement of *E. canadensis* by *E. nuttallii* (Barrat-Segretain et al., 2002; James et al., 2006). Interestingly, Barrat-Segretain (2005) concluded from an experiment that *E. nuttallii* seemed to be the better competitor of the two for light. Furthermore, data from The Netherlands and Central European waters suggested that the replacement of *E. canadensis* by *E. nuttallii* was especially pronounced in ditches in agricultural areas, where total nitrogen input was much higher (Knoben and Peeters, 1997; Király et al., 2007; Grudnik et al., 2014). A recent study showed that light and nitrogen jointly triggered the development of those phenotypic characteristics that makes *E. nuttallii* a more successful invader in eutrophic waters than *E. canadensis* (Szabó et al., 2019). These two factors (light availability and nitrogen) are strongly related to eutrophication. The responses to these factors separately have been well documented (light: Sand-Jensen and Madsen, 1991; Madsen and Sand-Jensen, 1994; Angelstein and Schubert, 2009; nutrients: Barrat-Segretain, 2004; James et al., 2006), but their combined impact has been investigated only on the phenotypic characteristics (Szabó et al., 2019).

In this study we go further. We hypothesize that small ecophysiological differences between the two *Elodea* species become more pronounced under increasing nitrogen concentrations and decreasing light conditions and this may contribute to the invasion success of *E. nuttallii*. The present study aims to evaluate this hypothesis by investigating the combined effects of light and nitrogen on the ecophysiological responses of the two *Elodea* species in an indoor experiment. Since both *Elodea* species may strongly modify light conditions if they are grown in co-cultures (Barrat-Segretain and Elger, 2004; Barrat-Segretain, 2005), both species were cultivated separately in order to exclude these effects.

## Methods

### Plant Collection, Preincubation

*E. nuttallii* shoots were collected from the Eastern Principal Channel, (N 47.860911°, E 21.382270°) and *E. canadensis* from the River Bodrog (N 48.172491°, E 21.363358°) Hungary. The selected apical shoots were preincubated for 18 days under experimental conditions. Shoots were set in five plastic boxes containing 20-L culture medium (Barko and Smart, 1985). The supply of phosphorus was ensured by adding K_2_HPO_4_ to the final concentration to 0.2 mg P L^−1^ and supply of micronutrients by adding TROPICA Supplier micronutrient solution. Final concentration of the solutions for nitrogen varied from oligotrophic (0.05 mg L^−1^), mesotrophic (0.25 mg L^−1^), eutrophic (0.5 mg L^−1^), and hypertrophic (2.5 and 5 mg L^−1^) among the treatments by adding NH_4_NO_3_ stock solution to the medium (Szabó et al., 2019). The culture medium was renewed every second day. The length of the shoots was reduced to 65 mm after preincubation. Subsamples of three initial shoots of each species from each nitrogen concentration was measured for fresh and dry weight (*W*_0_).

### Laboratory Experiment

*Elodea* plants (three shoots) were set on a plastic grid and placed in 2-L aquaria containing the culture media described above. All aquaria were put into a controlled temperature (23–25°C) water bath with renewing of the medium every second day. For both *Elodea* species, the five different N treatments were incubated in a 16:8 h L/D regime at five different light intensities varying from complete dark to well-illuminated conditions: 0, 10, 28, 80, and 180 µmol m^−2^ s^−1^ PAR photon flux density (Szabó et al., 2019). Illumination was carried out by 400 W metal halogen lamps and by using plastic gauze above the aquaria. Each treatment was replicated four times meaning that 200 aquaria were used. The plants were harvested after 12 days of incubation.

### Relative Growth Rate

Six leaves were taken from each shoot (18 leaves per aquarium) and divided into two portions. One portion was used for fresh weight and chlorophyll determination, the other for dry weight determination (*W*_leave_). For a single measurement per aquarium, dry weight of the three shoots and their roots of each aquarium were measured. Samples were dried at 65°C for 2 days. The root–shoot ratios were expressed on a dry-weight basis. The relative growth rate (RGR) of the plants was calculated as RGR = (ln*W_t_* − ln*W*_0_)/*t*, where *W*_0_ represents the initial and *W_t_* the final dry weight (in g) of the three plants in each aquarium and t is the growing time in days. The light compensation point for growth *I*_c_ (µmol m^−2^ s^−1^) was estimated according to Sand-Jensen and Madsen (1991). Weight loss in the dark (RGR_d_, 10^−3^ day^−1^) was measured for plants incubated in the dark for 12 days (0 µmol m^−2^ s^−1^) compared to initial weight (*W*_0_).

### Photochemical Efficiency

The actual photochemical efficiency of photosystem II (Φ_PSII_) was measured by means of chlorophyll fluorescence with MINI-PAM fluorometer (Walz, Germany). The measurement on the plants was carried out using the middle part of the apical shoots from each aquarium as described by Snel et al. (1998). The shoots were placed in a 25 ml glass tube and faced to the common end of the optical fiber of the fluorometer. The actual photochemical efficiency of photosystem II was calculated as Δ*F*/*F*_m_′ = (*F*_m_′ − *F*_s_)/*F*_m_′ with *F*_m_′ the maximal fluorescence and *F*_s_ is the steady-state fluorescence of the illuminated shoots (Genty et al., 1989). Steady-state fluorescence (*F*_s_) was achieved after exposure to actinic light for 10 min. Maximum-fluorescence under steady-state conditions (*F*_m_′) was determined by applying pulses of the saturating light when the actinic light was on (Marwood et al., 2001). The duration of the saturating light pulses was 500 ms and the pulses were given every 60 s. The average of three chlorophyll fluorescence measurements represented the photochemical efficiency of the plants in each aquarium.

### Chlorophyll Concentration

For a single measurement per aquarium, chlorophyll of the nine leaves from each aquarium was extracted in a test tube containing 6 ml 95% ethanol for 24 h at 4°C in the dark. Total chlorophyll concentrations were measured by spectrophotometry (T80+ Spectrometer, PG Instruments Limited, UK) and were calculated according to Lichtentaler (1987). Leaf chlorophyll concentrations were expressed on a dry-weight basis of the leaves (*W*_leave_).

### Elemental Composition

Samples were taken from the water at the end of the experiment. We first recorded pH and thereafter samples were filtered (mesh size 0.45 µm) and analyzed for NO_3_^−^–N, NO_2_^−^–N, NH_4_^+^–N (Technicon Auto Analyzer). At the end of the experiment nitrogen and carbon concentration of the dried plants had been grown at 10–180 µmol m^−2^ s^−1^ light intensity (160 samples) was analyzed by dry combustion using a Vario Max Cube elemental analyzer (Elementar GMBH, Germany).

### Statistical Analysis

Normality of the variables was checked by the Kolmogorov–Smirnov test. RGR, chlorophyll concentration of the leaves, root–shoot ratio and nitrogen concentration of the plants were all normally distributed (*P* > 0.05). Data of actual photochemical efficiency of photosystem II and C/N ratio were log-transformed for normality. A general linear model was used to test the significance of the factors (light, nitrogen, species identity) and their interactions on the variables. Residuals were checked for normality and homogeneity of variances was evaluated by Levene's test. Tukey post-hoc tests were used to evaluate which treatments differed from each other. Pairwise comparisons were used to test the variables for significant differences between species where the mean difference (MD) + standard error were indicated. All analyses were done using SPSS 16.0 software.

## Results

### Relative Growth Rate

**Figure 1:**
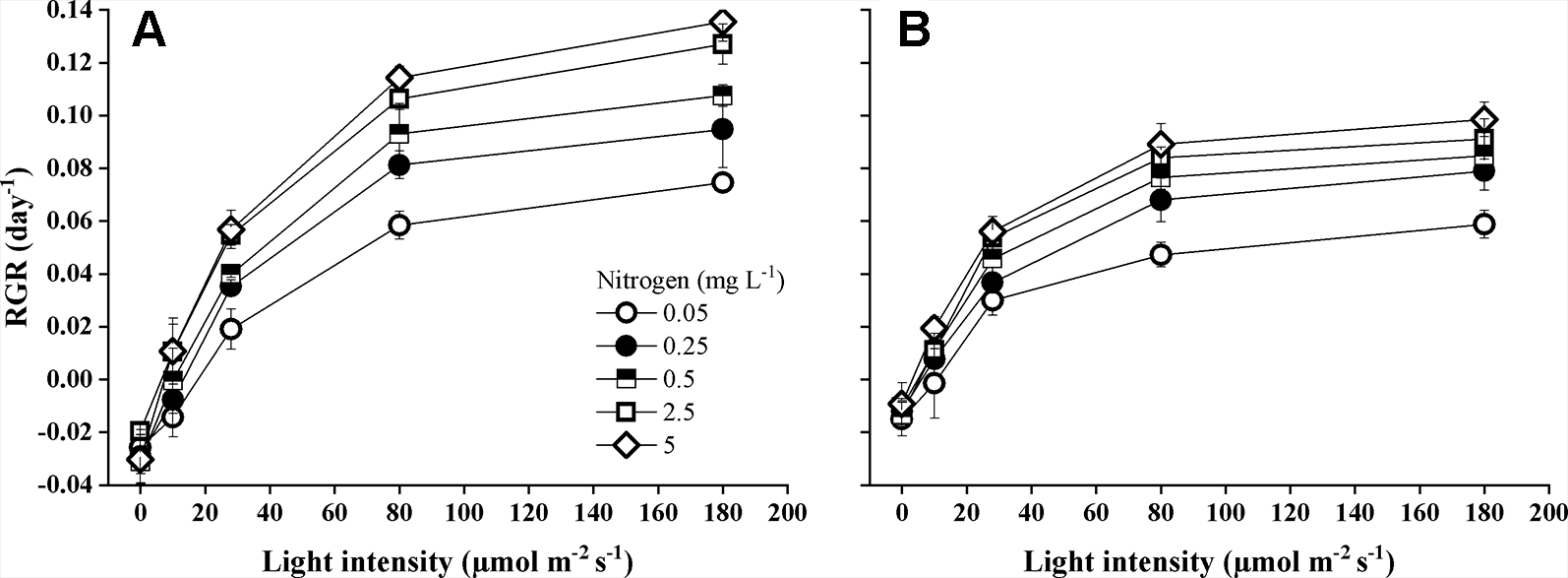
Relative growth rate (RGR) of *E. canadensis* **(A)** and *E. nuttallii* **(B)** cultures grown at different nitrogen concentrations and light levels (mean ± SD, *n* = 4).

**Figure 2:**
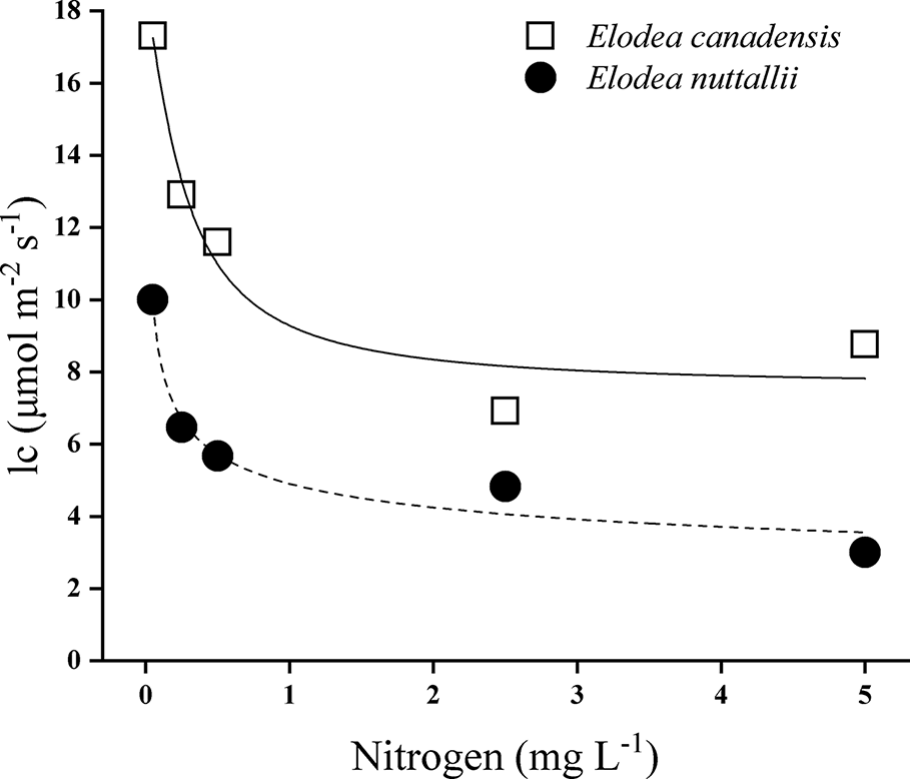
Light compensation point (*I*_c_) of *E. canadensis* and *E. nuttallii* cultures grown at different nitrogen concentrations. *I*_c_ values were estimated according to Sand-Jensen and Madsen (1991).

Species identity, nitrogen concentration, light intensity, and their interactions significantly influenced the RGR (**Table 1**). The RGR of both *Elodea* species increased with increasing light intensity and with increasing N concentration (**Figure 1**). Furthermore, pairwise comparisons showed that the differences between the species were statistically significant (**Table 1**). Growth seemed to be saturated above 80 µmol m^−2^ s^−1^ for *E. nuttallii* at all N concentrations except for the lowest one, and in contrast, light stimulated the growth of *E. canadensis* up to the highest light intensity (180 µmol m^−2^ s^−1^). The RGR measured at the highest light intensity was significantly higher for *E. canadensis* than for *E. nuttallii* (MD 0.025 ± 0.002 Pairwise comparisons *P* < 0.001). However, under low light levels (0–10 µmol m^−2^ s^−1^) *E. nuttallii* showed a significantly higher growth rate than *E. canadensis* (MD 0.012 ± 0.002 Pairwise comparisons *P* < 0.001). Weight loss in the dark differed significantly (pairwise comparisons *P* < 0.001) between the two *Elodea* species and was higher for *E. canadensis* than for *E. nuttallii* (MD 0.015 ± 0.002). The increase of N concentration from 2.5 to 5 mg L^−1^ did not cause differences in the growth rate of either species (**Figure 1**). The light compensation point (*I*_c_) decreased sharply with increasing N concentration and was significantly higher (MD 5.500 ± 2.131 pairwise comparisons, *P* = 0.033) for *E. canadensis* than for *E. nuttallii* (**Figure 2**).

**Table 1:** Analysis of variance of the relative growth rate (RGR), actual photosynthetic efficiency of PSII, chlorophyll concentration (Chl cc), root–shoot ratio of *Elodea* (*E. canadensis*, *E. nuttallii*) cultures grown in aquaria under different nitrogen concentrations in the water combined with different light intensities.

Source/Trait	*df*	Mean Square	*F*	Sig.
**RGR**				
Species	1	0.00	7.91	0.01
Light	4	0.10	1,739.81	<0.01
Nitrogen	4	0.01	112.24	<0.01
Light * Species	4	0.00	57.06	<0.01
Nitrogen * Species	4	0.00	4.72	<0.01
Light * Nitrogen	16	0.00	8.94	<0.01
Error	150	0.00		
**Photosynthetic efficiency**				
Species	1	0.01	32.21	<0.01
Light	4	0.86	1,903.57	<0.01
Nitrogen	4	0.06	134.30	<0.01
Light × Species	4	0.03	70.56	<0.01
Nitrogen × Species	4	0.00	9.44	<0.01
Light × Nitrogen	16	0.01	17.82	<0.01
Error	150	0.00		
**Chl cc**				
Species	1	1,975.04	544.42	<0.01
Light	4	892.66	246.06	<0.01
Nitrogen	4	2,112.38	582.28	<0.01
Light × Species	4	49.04	13.52	<0.01
Nitrogen × Species	4	77.45	21.35	<0.01
Light × Nitrogen	16	54.07	14.90	<0.01
Error	150	3.63		
**Root–shoot ratio**				
Species	1	0.02	144.82	<0.01
Light	4	0.12	841.85	<0.01
Nitrogen	4	0.02	161.74	<0.01
Light × Species	4	0.00	18.45	<0.01
Nitrogen × Species	4	0.00	15.86	<0.01
Light × Nitrogen	16	0.00	33.89	<0.01
Error	150	0.00		

### Photochemical Efficiency

**Figure 3:**
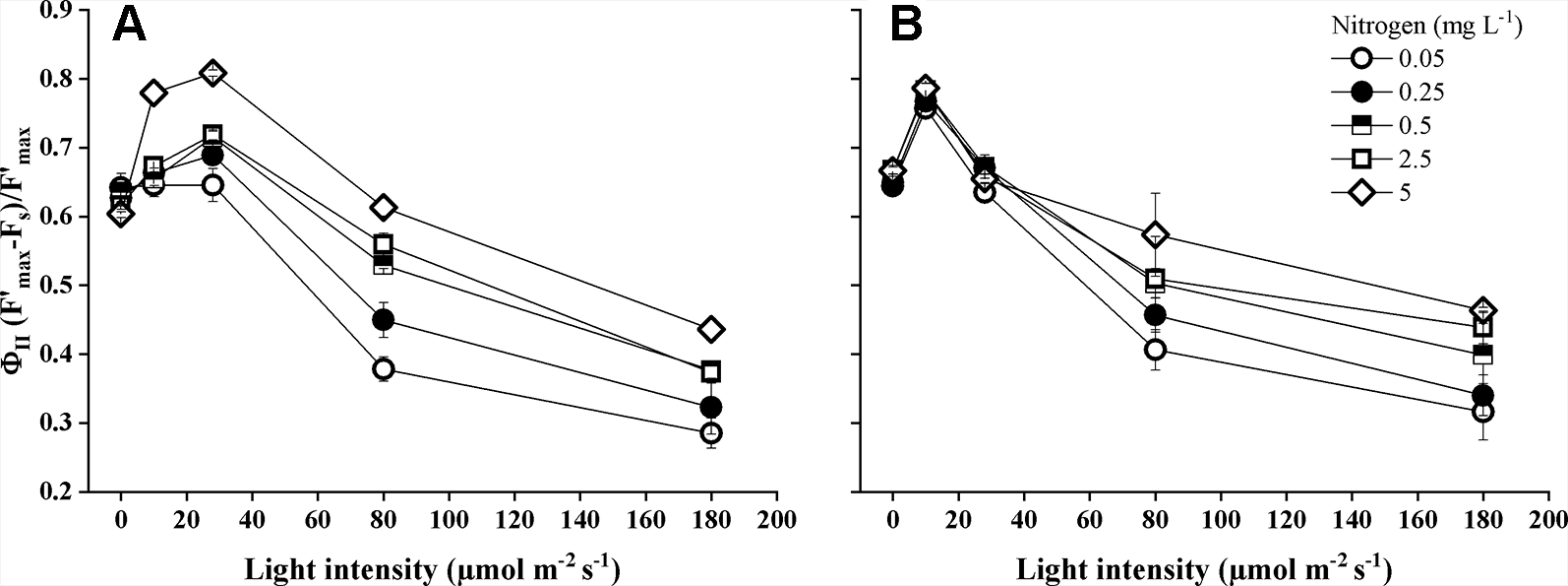
Actual photochemical efficiency of photosystem II (Φ_II_) of *E. canadensis* **(A)** and *E. nuttallii* **(B)** cultures grown at different nitrogen concentrations and light levels (mean ± SD, *n* = 4).

Light intensity, nitrogen concentration, and their interactions had a significant impact on the actual photochemical efficiency of photosystem II (Φ_PSII_) (**Table 1**). Both *Elodea* species showed the highest Φ_PSII_ at low and medium light levels (**Figure 3**, 0–28 µmol m^−2^ s^−1^) and the lowest Φ_PSII_ at the highest light level within each nitrogen concentration. Actual photochemical efficiency of photosystem II increased significantly at lower light intensities (*P* < 0.001) and at higher nitrogen concentrations (*P* = 0.022). Photochemical efficiency differed significantly (pairwise comparisons *P* < 0.001) between the two *Elodea* species and was higher for *E. nuttallii* than for *E. canadensis* (MD 0.017 ± 0.003). Under low light levels (0–10 µmol m^−2^ s^−1^), the difference for Φ_PSII_ between the two *Elodea* species was even higher (MD 0.063 ± 0.003) (**Figure 3**).

### Total Chlorophyll Concentration

**Figure 4:**
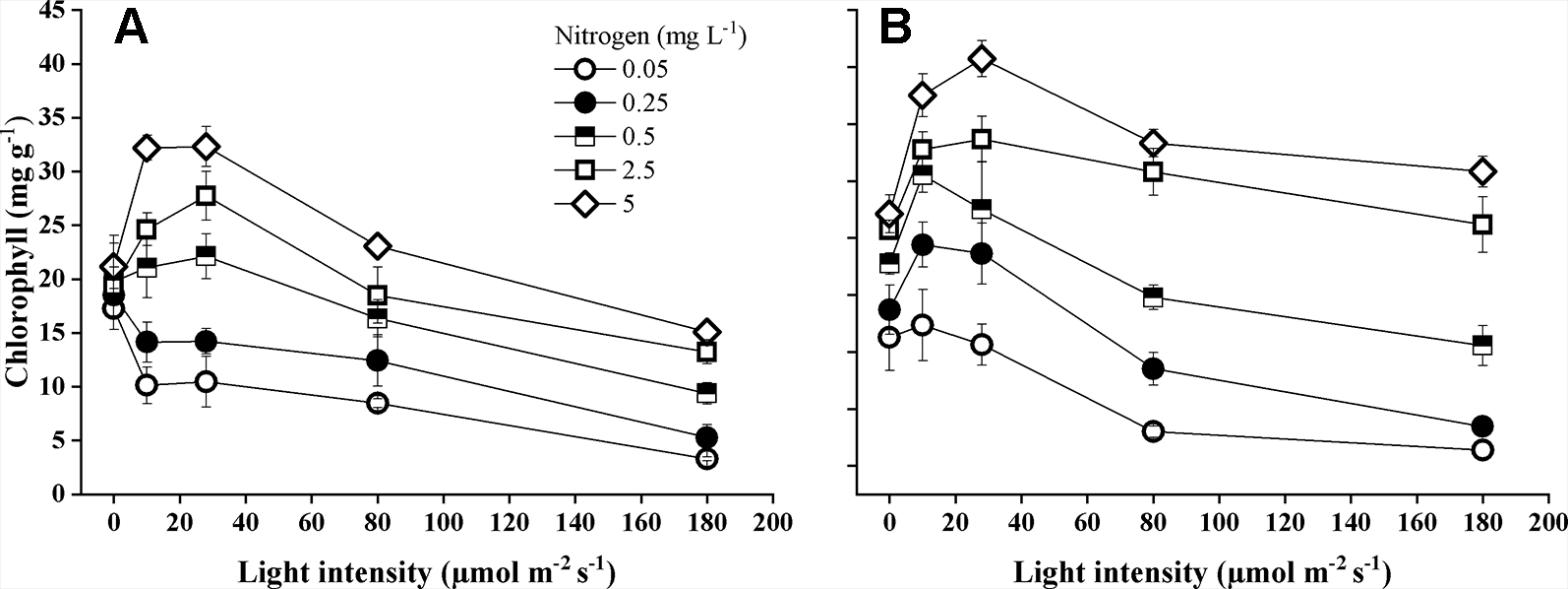
Chlorophyll concentration (μg mg^-1^) in the leaves of *E. canadensis* **(A)** and *E. nuttallii* **(B)** cultures grown at different nitrogen concentrations and light levels, (mean ± SD, *n* = 4).

Species identity, nitrogen concentration, light intensity, and their interactions significantly influenced total chlorophyll concentrations of the leaves (**Table 1**). Chlorophyll concentration was significantly higher (pairwise comparisons, *P* < 0.001) in *E. nuttallii* than in *E. canadensis* (MD 6.28 ± 0.27). According to the Tukey test, both species demonstrated that increasing light intensity resulted in significantly (*P* < 0.001) lower chlorophyll concentrations, but this was also depending on the N concentration (**Figure 4**). Increasing the light intensity, *E. canadensis* showed a much stronger drop in chlorophyll concentration than *E. nuttallii*. Lowering the nitrogen concentration significantly (*P* < 0.001) reduced chlorophyll concentration (**Table 1**) in both species (**Figure 4**).

### Root–Shoot Ratio

**Figure 5:**
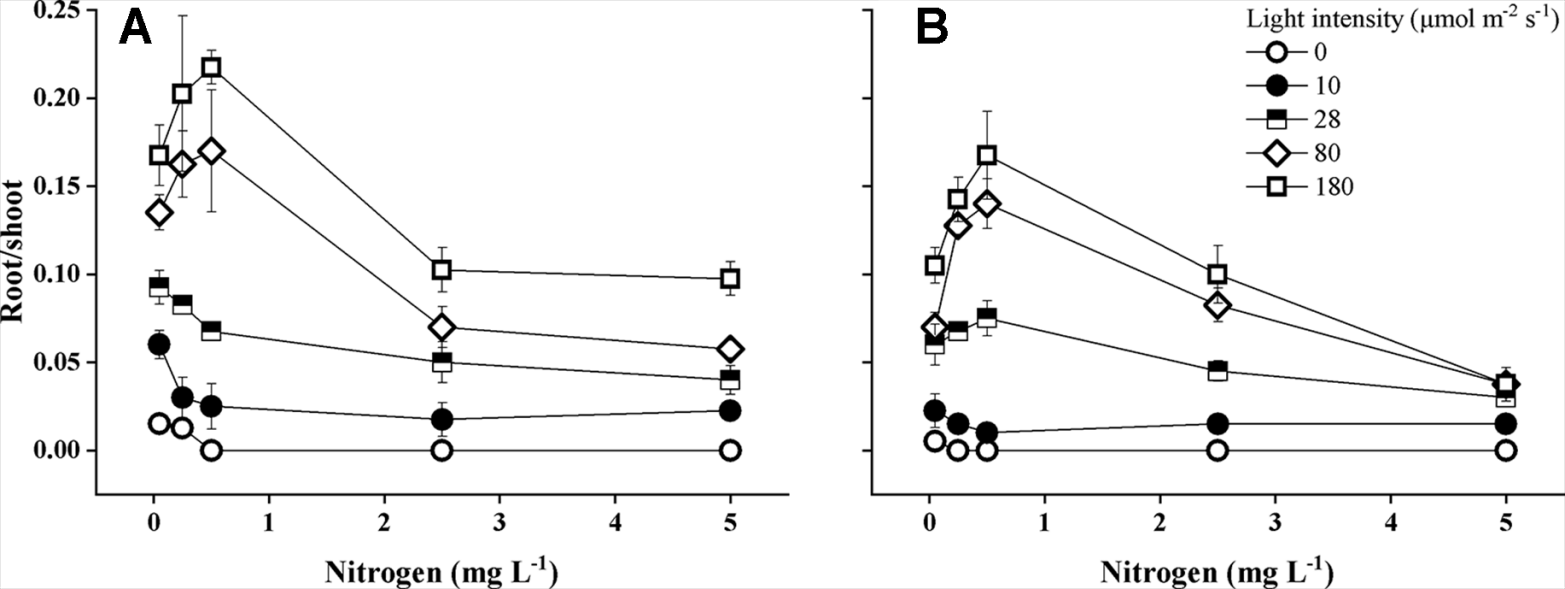
Root–shoot ratio of *Elodea canadensis* **(A)** and *E. nuttallii* **(B)** cultures grown at different nitrogen concentrations and light levels, (mean ± SD, *n* = 4).

Species identity, light intensity, and the statistical interaction between light intensity and N concentrations had a significant effect on root-shoot ratios (**Table 1**). Root–shoot ratios were the highest (0.17–0.22) at lower nitrogen concentrations (0.25–0.5 mg N L^−1^) and higher light intensities for both species (**Figure 5**). *E. canadensis* had significantly higher (pairwise comparisons, *P* < 0.001) root–shoot ratio than *E. nuttallii* (MD 0.021 ± 0.002). The difference in root–shoot ratios was even higher (MD 0.046 ± 0.006, *P* < 0.001) under the highest light level (180 µmol m^−2^ s^−1^).

### Chemical Composition of the Plants

**Figure 6:**
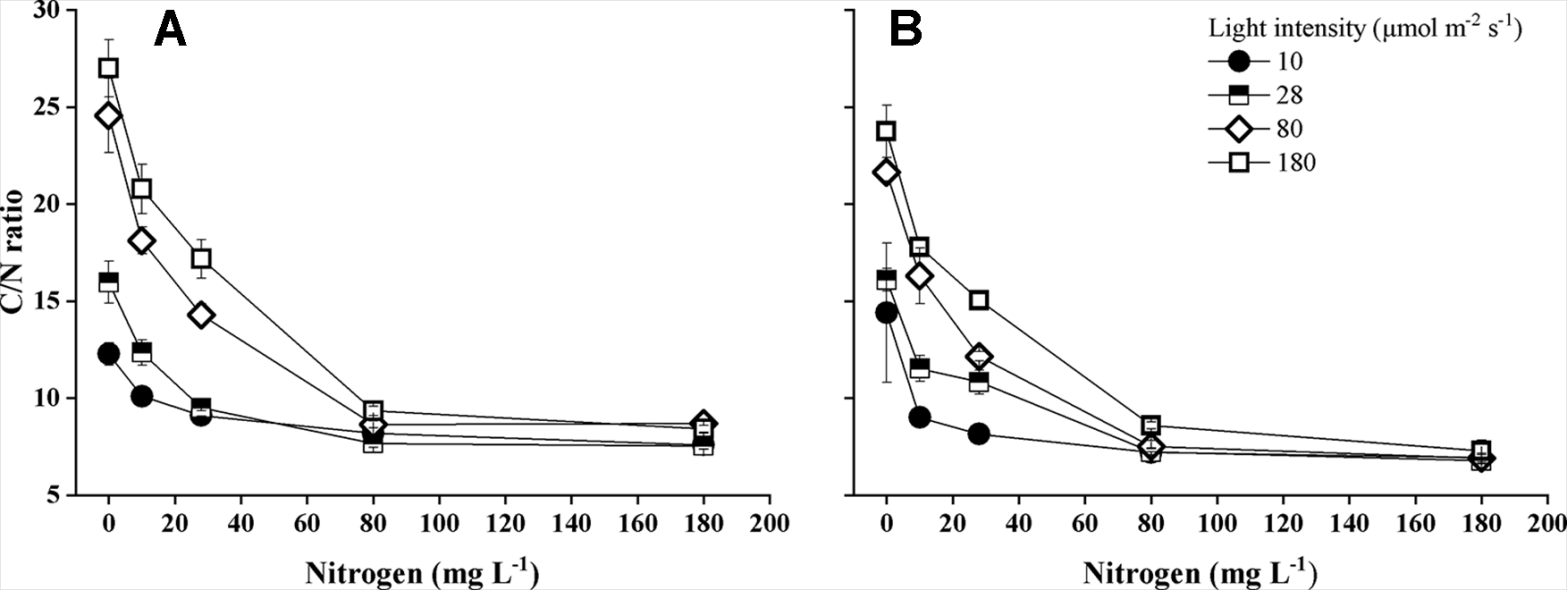
Carbon/nitrogen ratio (C/N ratio) of *E. canadensis* **(A)** and *E. nuttallii* **(B)** cultures grown at different nitrogen concentrations and light levels (mean ± SD, *n* = 4).

**Figure 7:**
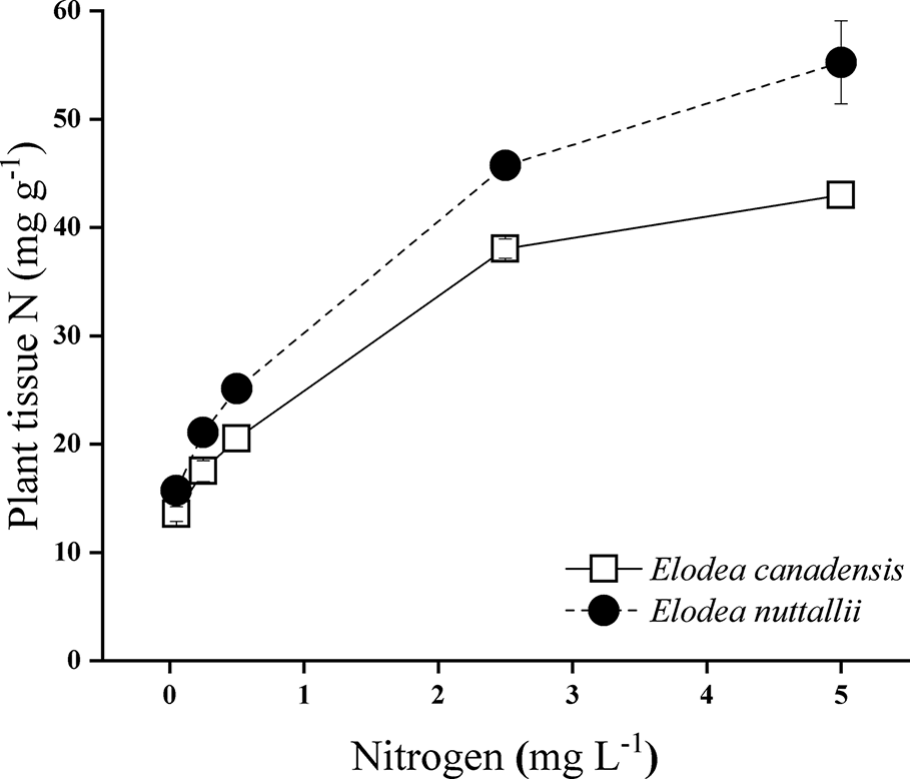
Plant tissue nitrogen concentration of *Elodea* species grown at different nitrogen concentrations at the highest light intensity (180 µmol m^−2^ s^−1^) (mean ± SD, *n* = 4).

Nitrogen concentration of the water, light intensity, and their interactions significantly influenced carbon/nitrogen ratio (C/N ratio) of the plants (**Supplementary Table 1**). C/N ratio of both *Elodea* species decreased with increasing N concentration and with decreasing light intensity (**Figure 6**). At the highest light intensity, tissue N concentration (mg N g^−1^) was significantly higher for *E. nuttallii* than for *E. canadensis* (MD 6.043 ± 0.342, pairwise comparisons *P* < 0.001) (**Figure 7**).

### Chemical Composition of Water

**Figure 8:**
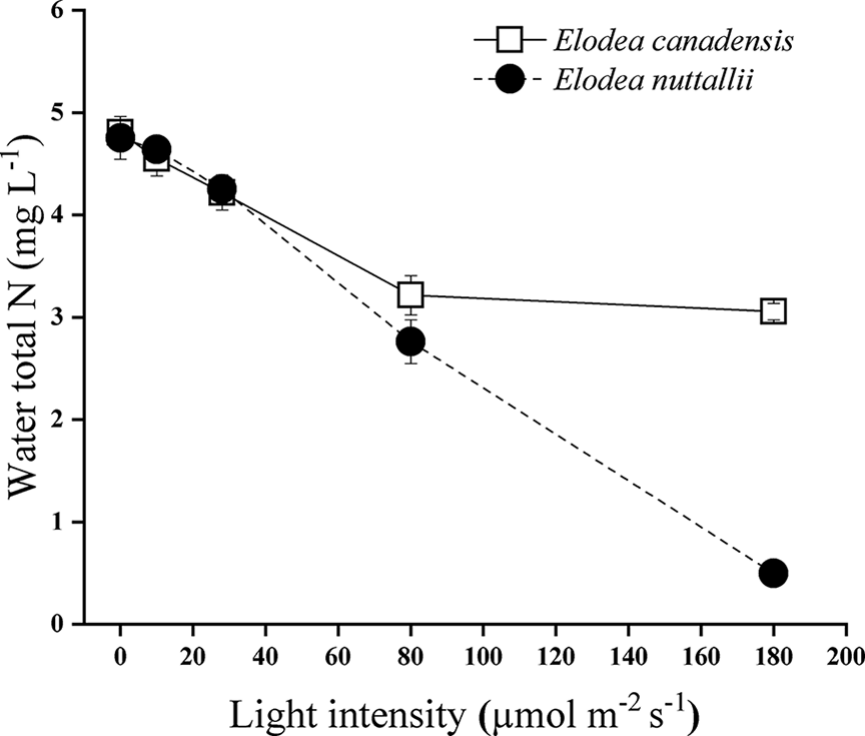
Total nitrogen concentration of the medium with *Elodea* species grown under 5 mg L^−1^ initial N concentration at different light levels (mean ± SD, *n* = 4).

Total nitrogen removal by the two examined *Elodea* species (expressed by a drop in total nitrogen concentration of the water) decreased with decreasing light intensities. The final N concentration was significantly lower with *E. nuttallii* than with *E. canadensis* (pairwise comparison *P* < 0.001; MD 0.451 ± 0.021) when initial concentration was higher than 0.25 mg N L^−1^. Under the highest light intensity and the highest initial nitrogen level (5 mg L^−1^), the difference in the final nitrogen concentration between the two species was even higher (pairwise comparison *P* < 0.001; MD 2.558 ± 0.057) (**Figure 8**). At the highest light intensity, *E. nuttallii* increased the pH more markedly (10.41) than *E. canadensis* (9.74). We found the highest at the highest light level. The pH decreased drastically at reduced light intensities.

## Discussion

### Interactive Effects of Light and Nitrogen

Interactive effects of various factors on plant growth have previously been observed more often for light and inorganic carbon, and for nitrogen and inorganic carbon (Madsen and Sand-Jensen, 1994; Madsen et al., 1998), light and temperature (Barko et al., 1982), light and nitrogen (Szabó et al., 2019), temperature and phosphorus (Peeters et al., 2013), and nitrogen and phosphorus (Cao et al., 2011; Li et al., 2016). In this study, light and nitrogen both affected the performance of the two *Elodea* species. Interactive effects of light and nitrogen were also obvious: increase in light stimulated growth twice as much under high nitrogen concentrations (2.5 and 5 mg L^−1^) than at lower N levels (0.05 mg L^−1^). Therefore, the same growth rate can be obtained under different conditions: reduced light conditions but with more nitrogen gave similar yields as lower nitrogen and more light.

### Light Compensation Point, Weight Loss in the Dark

The light compensation (*I*_c_) point of *E. canadensis* grown under optimal nitrogen supply in the present study closely approximates the results of Madsen and Sand-Jensen (1994) who kept the plants under an optimal carbon supply. They also found a sharp increase in the light compensation point under lowered inorganic carbon supply as with nitrogen levels in the present study. *E. canadensis* had a much higher light compensation point (*I*_c_) than *E. nuttallii*. This indicates that the latter species may survive better under more shaded conditions, such as turbid water, periphytic algae, or below a dense mat of floating plants (Angelstein and Schubert, 2009; van Gerven et al., 2015). Weight loss in the dark of *E. canadensis* was approximately two times higher than recorded by Madsen and Sand-Jensen (1994), which can be explained by dark respiration being lower at low temperatures. Interestingly, *E. nuttallii* showed lower weight loss in the dark than *E. canadensis*, and this might be an important indication that *E. nuttallii* can tolerate darkness for a longer period.

### Change in Chlorophyll Concentration

Since nitrogen is a crucial component of chlorophyll, it is not surprising that the total chlorophyll concentration of both *Elodea* species was strongly affected by the availability of nitrogen. Therefore, our results are in line with the findings of many authors (Szabó et al., 2005; Szabó et al., 2010; Zhao et al., 2010). In general, *E. nuttallii* had a higher chlorophyll concentration than *E. canadensis* and plants showed a peak in chlorophyll under rather low light conditions (10–28 µmol m^−2^ s^−1^) as was also found by Madsen and Sand-Jensen (1994). In complete darkness, however, both species lost some chlorophyll content since they did not receive sufficient energy to sustain chlorophyll synthesis and maintaining the photosynthetic apparatus for a long period is uneconomical (Raven, 1984).

### Change in Photosynthetic Efficiency

The actual photosynthetic efficiency of both *Elodea* species was slightly decreased under low nitrogen supply which is in line with the studies of Cruz et al. (2003) and Huang et al. (2004) and indicates that nitrogen deficiency causes damage to PSII reaction centers (Verhoeven et al., 1997; Lu et al., 2001). Furthermore, nitrogen is essential for protein synthesis in order to sustain or rebuild the photosynthetic apparatus (Evans, 1989). Under low light levels (0–10 µmol m^−2^ s^−1^) parallel to the RGR results, *E. nuttallii* showed marginally but significantly higher actual photosynthetic efficiency than *E. canadensis*. Higher Φ_PSII_ efficiency of *E. nuttallii* can be taken as a characteristic related to shade tolerance only in combination with the lower lc which the primary factor here. Remarkably, after twelve days of incubation in total darkness, both *Elodea* species still showed significant photosynthetic activity indicating that if oxygen is not limited, they can tolerate dark conditions for extended periods.

### Morphological and Ecophysiological Strategies

**Figure 9:**
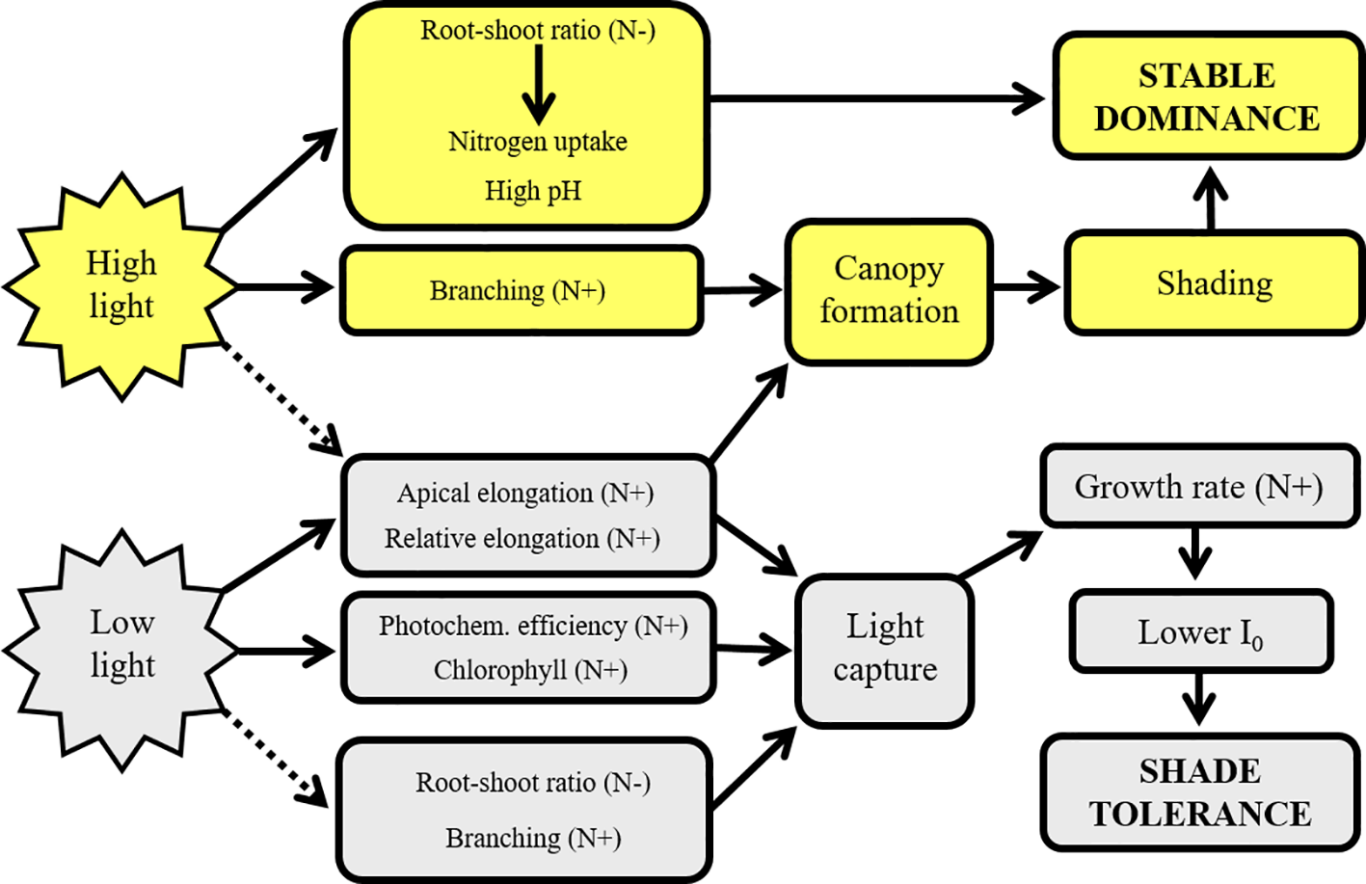
The effect of light levels and nitrogen on the ecophysiological and phenotypic traits of *Elodea nuttallii* resulting in invasion success. Solid lines represent stimulation, dashed lines represent inhibition processes. N+ and N− indicate the stimulating or inhibitory impacts of increased nitrogen supply, *I*_0_ light compensation point.

Both *Elodea* species showed a high ability to acclimate to various light and nutrient levels, and they shared many similarities in their ecophysiological and morphological responses (**Figure 9**). Under high nitrogen levels the plants seem to invest more energy in photon capture than in nutrient uptake as evidenced by the reduced root-shoot ratio (James et al., 2006), light compensation point, increased chlorophyll concentration and photochemical efficiency which is in line with the studies of Li et al. (2016) and Gautam et al. (2016). An alternative explanation could be that plants invest less in root biomass since less below-ground biomass is necessary for nutrient uptake in N rich water (Madsen and Cedergreen, 2002). At low light levels, similarly as high nitrogen levels, the plants also redirected resources towards a more efficient photon capture rather than nutrient uptake as proved by the increase in the chlorophyll concentration in the leaves together with a higher actual photochemical efficiency of PSII, increased shoot elongation per unit biomass and reduced allocation to root formation and reduced nutrient removal (**Figure 9**). At low light levels, *E. nuttallii* the stronger invader showed drop in branching.

Increase in chlorophyll concentration with lowering light is well in line with the results of Angelstein and Schubert (2009). In our study, both morphological (root–shoot ratio) and ecophysiological (actual photochemical efficiency of PSII, chlorophyll concentration) changes were similar to those observed in response to decreased light intensities caused by shading from floating vegetation which has been found in other submerged macrophytes (Janes et al., 1996; Forchhammer, 1999; Lu et al., 2013).

### Displacement Mechanisms

Our laboratory study showed that *E. canadensis* had a slightly higher growth rate than *E. nuttallii*, supporting the view that both species responded similarly to changing light and N-levels. The directions of the differences were always similar; however the magnitude was different (**Supplementary Table 2**). The replacement of *E. canadensis* by *E. nuttallii* indicates that there must be differences between both species. From our laboratory experiment we are not able to infer the competitive abilities of the two *Elodea* since we had no mixed cultures (McCreary, 1991). However, it is evident that in a competition experiment, the competition itself may mask relevant mechanisms but also small differences between the two species. The data of tissue N concentration and the decrease in nitrogen concentration in the water indicated that under higher light intensity *E. nuttallii* has much stronger nitrogen removal capacity than *E. canadensis* (**Figure 8**, **Supplementary Table 2**). This trait may especially advantageous in waters with suboptimal nitrogen concentration. Furthermore, *E. nuttallii* exhibited less reduction in biomass and showed lower light compensation point than *E. canadensis* and seemed thus more shade tolerant (**Supplementary Table 2**). In hypertrophic ditches and ponds, thick layers of floating algal mat reduce light conditions (Hillebrand, 1983) in spring whereas in summer floating vegetation may cause dense shade on submerged plants reducing their growth (Scheffer et al., 2003; van, Zuidam and Peeters, 2013; van Gerven et al., 2015). In addition, at high trophic levels *E. nuttallii* has much less periphytic algal biomass than *E. canadensis* (James et al., 2006) thus the new invader may have an even greater advantage for light capture.

Our former results pointed out that *E. canadensis* tends to produce dense canopy with numerous branches even under low nitrogen and light levels. Thus, *E. canadensis* shows less apical growth that might be a disadvantage in the competition for light with *E. nuttallii*. On the contrary, *E. nuttallii* invests much more on apical shoot elongation and thereby gain a better position for light capture (Szabó et al., 2019) (**Figure 9**, **Supplementary Table 2**). Under low light levels, *E. nuttallii* is able to elongate much faster due to its higher elongation and lower branching degree abilities and lower light compensation point (**Figure 9**). Thus, the shoots of *E. nuttallii* are able to achieve optimal light conditions sooner. Near to the water surface under high trophic level, they can form a dense canopy (Kuni, 1984) due to their increased branching degree, resulting in a strong shading for other submerged plants (**Figure 9**). Therefore, under hypertrophic conditions stands of *E. nuttallii* may develop sooner than that of *E. canadensis* and the stronger invader can sustain its stable dominance not only against other submerged plants but against algae and floating vegetation as well (Szabó et al., 2010).

Field observations showed that the replacements of *E. canadensis* by *E. nuttallii* occurred under hypertrophic conditions with nitrogen concentrations above 2 mg L^−1^ (Knoben and Peeters, 1997; van Zuidam and Peeters, 2013; Kočic et al., 2014). Actually, in this study, the niche for light requirement of *E. nuttallii* was narrower than that of *E. canadensis*. Therefore, our results partly contradict the idea of Higgins and Richardson (2014) who concluded that stronger invaders have broader physiological niches. However, this kind of adaptation to achieve fitness and invasiveness was more optimal under shaded conditions. These ecophysiological differences between the two species provide insights that could improve the understanding of the mechanisms of invasion processes under varying light and nutrient levels (**Supplementary Table 2**, **Figure 9**).

## Data Availability Statement

The datasets generated for this study are available on request to the corresponding author.

## Author Contributions

SS designed and performed the experiments. PN analyzed the chemical composition of the plants. SS, EP, and BL analyzed the data. SS, BL, GB, SV, and EP wrote the manuscript.

## Funding

This work was supported by GINOP grant (2.3.2-15-2016-00019) and OTKA grants (FK127939 and KH129520). BL was supported by the Bolyai János Research Scholarship of the Hungarian Academy of Sciences and by the New National Excellence Program of the Ministry of Innovation and Technology (UNKP-19-4-DE-193).

## Conflict of Interest

The authors declare that the research was conducted in the absence of any commercial or financial relationships that could be construed as a potential conflict of interest.
