# Supplementary material for: The Ecophysiological Response of Two Invasive Submerged Plants to Light and Nitrogen
Source: Front Plant Sci. 2020 Feb 7;10:1747. doi: 10.3389/fpls.2019.01747 (PMC7019179; doi:10.3389/fpls.2019.01747)
Supplement: Supplementary file 1 [file Table_1.doc]

**Electronic Supplementary Material 1. Analysis of variance of the carbon to nitrogen ratio (C/N ratio) of *Elodea* (*E. canadensis*, *E. nuttallii*) cultures grown in aquaria under different nitrogen concentrations in the water combined with different light intensities. Data were log transformed.**

| **Source/Trait** | **df** | **Mean Square** | **F** | **Sig.** |
| --- | --- | --- | --- | --- |
| **C/N ratio** |  |  |  |  |
| Species | 1 | 0.06 | 94.83 | <0.01 |
| Light | 3 | 0.31 | 468.40 | <0.01 |
| Nitrogen | 4 | 0.88 | 1 324.57 | <0.01 |
| Light * Species | 3 | 0.01 | 10.66 | <0.01 |
| Nitrogen * Species | 4 | 0.00 | 4.75 | <0.01 |
| Light * Nitrogen | 12 | 0.03 | 44.28 | <0.01 |
| Error | 120 | 0.00 |  |  |
