# Supplementary material for: The Ecophysiological Response of Two Invasive Submerged Plants to Light and Nitrogen
Source: Front Plant Sci. 2020 Feb 7;10:1747. doi: 10.3389/fpls.2019.01747 (PMC7019179; doi:10.3389/fpls.2019.01747)
Supplement: Supplementary file 2 [file Table_2.doc]

**Electronic Supplementary Material 2.** Differences in ecophysiological and phenotypic traits between *E. canadensis* (*Ec*) and *E. nuttallii* (*En*). *Data by Szabó et al. (2018).

| **Trait** | ***Ec*** |  | ***En*** |
| --- | --- | --- | --- |
| Chlorophyll |  | **<** |  |
| Photochemical efficiency |  | **<** |  |
| Growth in shade |  | **<** |  |
| weight loss in dark |  | **>** |  |
| Light compensation point |  | **>** |  |
| Root-shoot ratio |  | **>** |  |
| Nitrogen uptake |  | **<** |  |
| Apical elongation* |  | **<** |  |
| Shoot relative elongation* |  | **<** |  |
| Branching in shade* |  | **>** |  |
